# A simple method for activating the platelets used in microfluidic platelet aggregation tests: Stirring-induced platelet activation

**DOI:** 10.1063/1.4972077

**Published:** 2016-12-15

**Authors:** Hoyoon Lee, Gyehyu Kim, Chaeseung Lim, ByoungKwon Lee, Sehyun Shin

**Affiliations:** 1 School of Mechanical Engineering, Korea University, Seoul 136-701, South Korea; 2 Department of Laboratory Medicine, College of Medicine, Korea University Guro Hospital, Seoul 152-703, South Korea; 3 Department of Internal Medicine, College of Medicine, Yonsei University Gangnam Severance Hospital, Seoul 135-720, South Korea

## Abstract

High-shear stimulation is well known as one of the key factors affecting platelet activation and aggregation, which can lead to the formation of a thrombus. In one of our previous studies, we introduced migration distance-based platelet function analysis in a microfluidic system. In this study, we set out to examine the effects of stirring on shear-induced platelet activation and aggregation in a chamber system by using a rotating stirrer. We found that the rotating stirrer caused not only rotational shear flow but also a strong radial secondary flow. The latter flow led to efficient mixing in the chamber. Moreover, the rotational flow led to the generation of shear stress, the magnitude of which can be controlled to activate the platelets. Activated platelets tend to aggregate themselves. The maximum platelet aggregation was observed at a critical shear rate of 3100 s^−1^, regardless of the stirrer shape. Furthermore, the time taken to attain maximum aggregation was significantly shortened when using a wide stirrer (30 s) instead of a narrow one (180 s). When using a flat stirrer, the non-uniform shear field in the chamber system was resolved with the radial secondary flow-induced mixing; thus, most of the platelets were homogenously activated. The stirring-induced platelet activation mechanism was experimentally confirmed in a microfluidic system for a platelet aggregation test while monitoring the migration distance until the microfluidic channel is occluded. Our findings indicate that the present system, consisting of a rotating stirrer and a confined chamber, provides effective shear stimulation for activating platelets and inducing platelet aggregates.

## I. INTRODUCTION

Platelets are important blood cells whose function is closely related to hemostasis. Platelet aggregation is caused by platelet activation, which is associated with direct contact with agonists, such as subendothelial collagen,^1–3^ as well as an abnormally high shear stress.^4–7^ A recent study showed that mechanical shear stimulation plays a dominant role in platelet aggregation and thrombus growth.^6,8^ It is also known that a pathological shear stress of 8 Pa is sufficient to activate platelets and induce the formation of a thrombus.^8–10^

With advances in microfluidic technology, many methods have been proposed for realizing shear-induced platelet activation (SIPA) tests.^10–16^ The PFA-200 (Siemens, Germany) is one of the most widely used clinical diagnostic instruments for SIPA tests that uses a high shear rate ($\dot{\gamma }$ > 5000 s^−1^).^12,13,15^ In this device, a blood sample flows through a capillary (d = 200 *μ*m), in which platelets may be activated. Another example of a microfluidic SIPA test is the T-tas (Zacros, Japan),^17^ which consists of agonist-coated multi-capillaries, on which platelets tend to adhere and aggregate under elevated shear conditions (1000 < $\dot{\gamma }$ < 2000 s^−1^).^11,14^

However, there are several limitations with the shear-generating method, centering on the pressure-driven flow through either capillary or multi-channels. Notably, the pressure-driven flow through a capillary has a parabolic velocity profile, which yields a non-uniform shear stress distribution over the cross-sectional area. Thus, platelets flowing through capillaries would be subjected to non-homogeneous shear stress profiles, which may result in incomplete activation of the platelets. Furthermore, a recent study^18^ raised an issue that insufficient shear rate generation might be the cause of failure of platelet function tests to guide personalized antithrombotic medication.

To overcome these limitations, there is a need for a simple and efficient shear-generating mechanism. In our previous studies, we demonstrated that a shearing mechanism with a stirrer could provide an adjustable uniform shear rate (i.e., stress) to activate the platelets.^19,28^ Our study elucidated that shear-induced activated platelets adhered to collagen coated surfaces and formed aggregates. Subsequently, the collagen coated area in the channel was blocked with platelets and other blood cells, and the pressure-driven flow stopped. Thus, the platelet function under regulated shear conditions was successfully quantified using the migration distance (MD) through a microchannel under vacuum pressure.

However, although the shear-generating stirrer has been conceptually accepted for observing activated platelets, it has not been fully understood with respect to the fluid dynamics associated with the stirring-induced shear stress and the fluid flow. Therefore, the present study set out to investigate the mechanisms of stirrer-induced shear generation and their effect on platelet activation and aggregation. Finally, the stirring-induced platelet activation mechanism was applied and verified for a platelet aggregation test in a novel microfluidic system.

## II. MATERIALS AND METHODS

### A. Sample preparation

Whole human blood was collected in sodium citrate (BD Vacutainer, NJ, USA) by means of venipuncture. Blood samples were obtained from four healthy volunteers who were not taking any medication. Platelet-rich plasma (PRP) was extracted from the whole blood (WB) by centrifuging at 140 g for 15 min. All of the tests were completed within 4 h to minimize the risk of time-dependent platelet malfunction. Furthermore, the platelets were optionally stained with DIOC_6_ (Sigma-Aldrich, Ontario, Canada) to distinguish them from other blood cells in a fluorescent-field microscope photograph. For staining with DIOC_6_, platelets obtained from PRP were washed thrice with phosphate buffered saline (PBS), and the washed cells were suspended in PBS. Then, diluted DIOC_6_ in PBS was added to the cell suspensions, and the final concentration of DIOC_6_ was 0.5 *μ*g/ml. After incubation for 1 h at room temperature, the cells were washed five times with PBS. To prepare negative blood (NB), the PRP was centrifuged again at 380 g for 15 min. The upper plasma with depleted platelets, which is called platelet-poor plasma (PPP), was re-mixed at the hematocrit level of the donor.

### B. Shear rate in rotating stirrer-chamber system

**FIG. 1.**
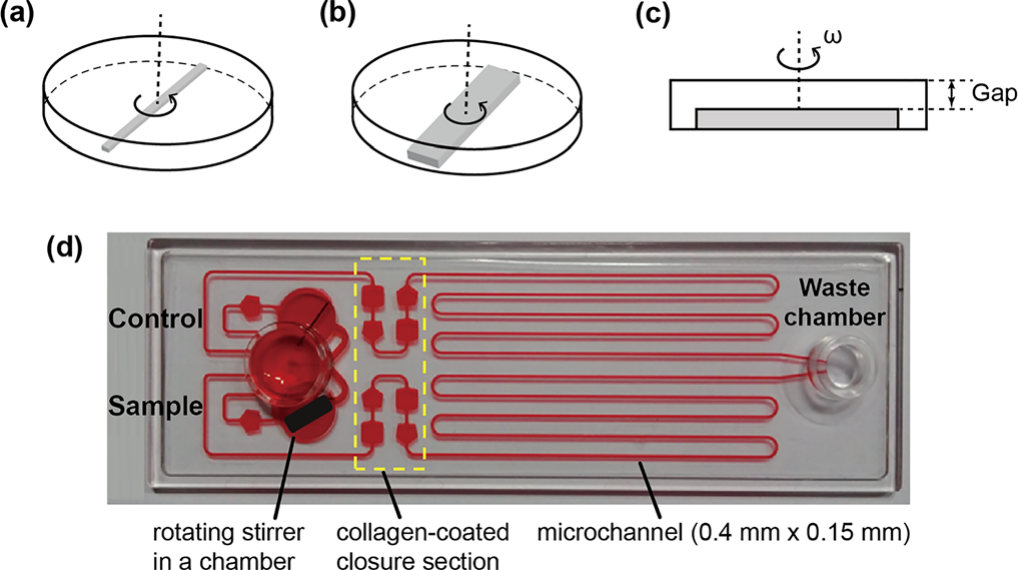
(a) Schematic of the chamber system with a rotating stirrer, using a simple one-bar stirrer. (b) Schematic of the chamber system with a rotating stirrer, using four-bar stirrers. (c) Cross-sectional view indicating the rotational speed and gap between the stirrer top and chamber top. (d) A photograph of the microfluidic system consisting of an inlet chamber, a pair of sample chambers, a pair of collagen-coated closure sections, and a pair of main lanes connected to a common outlet.

Figure 1 is a schematic diagram of a stirrer in a sample chamber, which resembles a parallel-disc rheometry. As shown in Figure 1(d), the working principle for the platelet function assay using the microfluidic system can be found elsewhere.^19^ The microfluidic system enables the activation of platelets at adjustable shear rates and allows adhesion of the activated platelets to specific microchannel areas. The system consists of a micro-chamber, a main microchannel section (150 *μ*m in height and 0.8 mm in width), a solenoid valve, and a syringe pump. The chamber for the platelet test was 10 mm in diameter. The different heights of the chamber were prepared to set a specified fluid gap (*h*) from 0.25 to 1.32 mm. The bar stirrers are made of SS400, which can be easily magnetized when a magnetic field is applied. These bar stirrers were fabricated using an etching process, with a standard deviation in stirrer dimensions of less than 0.1 mm. The typical dimensions of a rectangular bar stirrer (so called 1×) are 1 mm in width, 2 mm in height, and 7 mm in length, respectively. To increase the width of the stirrer (*w_stirrer_*), up to five individual bar stirrers were bonded in parallel (5× bar stirrer).

After stirring for a certain period of time, the sheared blood samples were driven by vacuum pressure. As a large dead volume chamber (25 ml) was connected to the microfluidic system, there was no apparent change in the vacuum pressure during the entire test period. At the entrance of the microchannel, the bottom surface was coated with collagen, a strong agonist, to promote the adhesion of platelets. While flowing through the collagen-coated microchannel, activated platelets tended to aggregate and adhere to the surface. The adhesion and aggregation of platelets on the bottom surface of the microchannel would gradually result in a reduction of the cross-sectional area of the microchannel; eventually, the blood flow would stop. The distance from the entrance to the final stop position of the blood sample is defined as the migration distance (MD), which represents the response of the platelets to shear activation as well as their aggregation.

The rheological shear flow of parallel discs is well-defined.^20^ The corresponding shear rate for a given radial position in the parallel discs can be derived from the equations of motion, as follows:

$$\dot{\gamma }(r)=\frac{r\omega }{h},$$ (1)

where *r*, *ω*, and *h* are the radial distance, angular velocity, and flow gap, respectively. As illustrated by Eq. (1), shear rates are radius-dependent for the rotating parallel discs.

Notably, although the directional velocities other than the peripheral one were neglected, in an actual flow, there is a secondary flow in the radial direction (Bödewadt layer), which is inward near a stationary disc and outward near a rotating disc.^21–24^ This implies that the secondary flow facilitates sufficient mixing and homogeneous platelet activation under a rotating shear flow. In this case, the average shear rate ($\dot{\gamma }$_avg_) over the cross-sectional area of the parallel discs was utilized as a representative value to determine the rheological conditions in the rotating stirrer

$${\dot{\gamma }}_{avg}=\frac{\iint r\dot{\gamma }(r)drd\theta }{\iint rdrd\theta }=\frac{2R\omega }{3h}.$$ (2)

### C. Flow visualization

To obtain a hydrodynamic understanding of stirring in a circular chamber, a flow-visualization system was prepared. The system consists of a transparent circular chamber with a lid, a dye-injection system, and a high-speed camera. Dye was injected though a hole in the lid, and the streamlines were captured while the stirrer rotated. For convenience, the size of the chamber used for the flow visualization was greater than that used for the platelet aggregation test with 20 mm in diameter and 5 mm in height.

### D. Platelet aggregation tests

For the platelet-activation test, 150 *μ*l of PRP or whole blood was pipetted into the sample chamber. Shear stimulation was applied by rotating the stirrer in the samples for a specific shearing time ranging from 0 to 180 s. The rotational speed of the stirrer was remotely controlled using a magnet mounted on the rotor. A 10 μL sample of sheared PRP or whole blood was immediately smeared onto a cleaned glass slide and then observed with a microscope. The smeared platelets or blood cells were observed in both the optical and fluorescent fields.

### E. Image-based analysis of platelet aggregation

Activated platelets tend to aggregate, and the sizes of the aggregates are proportional to the degree of activation.^25–27^ That is, the size or area of the platelet aggregates reflects the degree of platelet activation. The average area of the platelet aggregates, taken from more than 20 plural microscopic images, was measured by image analysis utilizing the *ImageJ* software (www.imagej.net). The area of the identified platelets and their aggregates was determined in terms of the number of pixels and converted into square micrometers (*μ*m^2^). A threshold area of 50 *μ*m^2^, which is the sum of the areas of seven platelets, was selected as the threshold value to prevent overlapping in the analysis of platelet aggregation.

### F. Platelet activation test in microfluidic system

The stirring-induced platelet activation mechanism was verified using the microfluidic system as shown in Figure 1(d). This microfluidic system, which adopted the same operating principle of our previous study,^19,28^ was significantly revised and enhanced with precision design. The basic mechanism for quantifying platelet function is identical to that for a previous study using migration distance (MD), which is defined as the distance from the entrance to the position at which the blood flow finally stops, as an effective parameter for platelet function including activation, adhesion, and aggregation.^19,28^ A microchip was configured with an inlet chamber, a pair of sample chambers, a pair of collagen-coated closure sections, and a pair of main lanes connected to a common outlet. The microchip was formed from a pair of cyclic olefin copolymer (COC) plates. The channels were engraved into the upper plate and it was firmly bonded using double-sided adhesive tape (thickness = 40 *μ*m) with a low flat plate. The dimensions of the main channel were 400 *μ*m × 150 *μ*m (width × height).

300 *μ*l of whole blood was injected into an injection chamber. When the shearing chamber had completely filled with blood, the applied pressure was returned to atmospheric pressure to halt the flow. Shear stimulation was selectively applied to the sample chamber alone at 3100 s^−1^ for 30 s. To prevent any unexpected blood migration caused by the stirring, capillary valves with a pair of pentagonal chambers were designed in front of a shearing chamber, as shown in Figure 1(d). The pair of pentagonal chambers was designed as a capillary valve, which would prevent the fluid from flowing in an unwanted direction through the microchannel. After the shear stimulation, the flow of the two blood samples was driven by a specified vacuum pressure (Δp = 1.2 kPa), applied by operating a solenoid valve. With adding the dead volume chamber, the applied vacuum pressure remained constant during the whole period of a test. The activated platelets in the sheared blood began to adhere to the collagen-coated chambers and thus increased the flow resistance through platelet aggregation. Thus, the velocity of sheared blood gradually decreased and eventually stopped, while the control blood to which no shear was applied flowed further than the sheared sample.

## III. RESULTS AND DISCUSSION

**FIG. 2.**
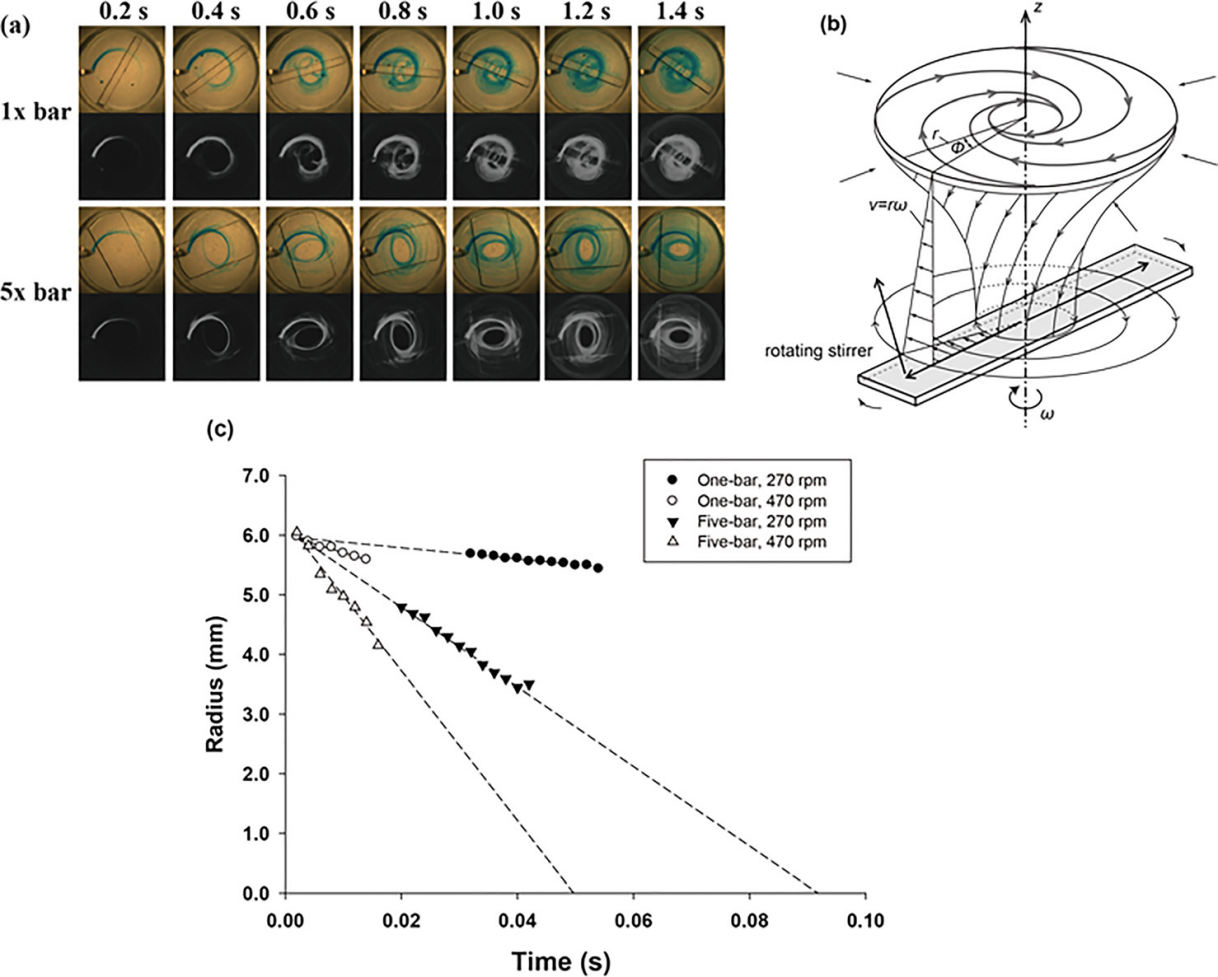
(a) Streamlines, observed from above, for different stirrer shapes: one-bar and five-bar. The streamlines in the second row in each case were converted into grayscale by using *ImageJ.* The sampling interval of the pictures was 200 ms. (b) Schematic view of the rotating flow pattern near a stationary disc with the velocity in theta direction, *v*. The stirrer-driven rotational flow generates the secondary flow in the inward direction of the radial axis. (c) Radial migration of streamlines injected from the top lid at r = 6 mm.

As a result, Figure 2 shows the streamlines observed from above and the schematic flow structures in a circular chamber with a rotating stirrer. The dyed particles injected from the top of the lid gradually moved inward, forming a spiral, as shown in Fig. 2(a). When the dyed particles reached the central area at the bottom of the chamber, they immediately spread throughout the entire chamber because of the action of the rotating stirrer. Then, these particles moved upward to the top edge. Consequently, the dyed particles completed a round trip with a downward spiral flow and an instant centrifugal flow. During the round trip of a particle, it experiences various shear rates with respect to its radial location (*r*) and angular velocity (rpm), as defined in Eq. (1). Thus, the representative shear rate that a particle experiences would be the average shear rate, as defined in Eq. (2).

The time required for the round trip of a particle was dependent on both the rpm and the width of the stirrer. As shown in Figure 2(c), the wider the stirrer and the higher the rpm, the shorter the time required for radial migration of the dyed particles. For instance, by extrapolating the lines for 470 rpm and 5× bar, we can determine that a dyed particle injected at r = 6 mm will arrive at the center of the chamber within 0.05 s. Thus, the round trip takes approximately twice as long as a one-way trip at the most, which is about 0.1 s. Considering that the typical shearing time was 30 s, a single particle would complete a total of 300 round-trips, which would generate sufficient mixing with homogenous shearing. That is, particles in a chamber migrate in the radial direction and thus experience different shear rates according to the radial position, as discussed in Eq. (1). Therefore, although a stirrer in a chamber generates a non-uniform shear field, it produces uniform activation of all the platelets with sufficient and fast mixing in a blood sample.

**FIG. 3.**
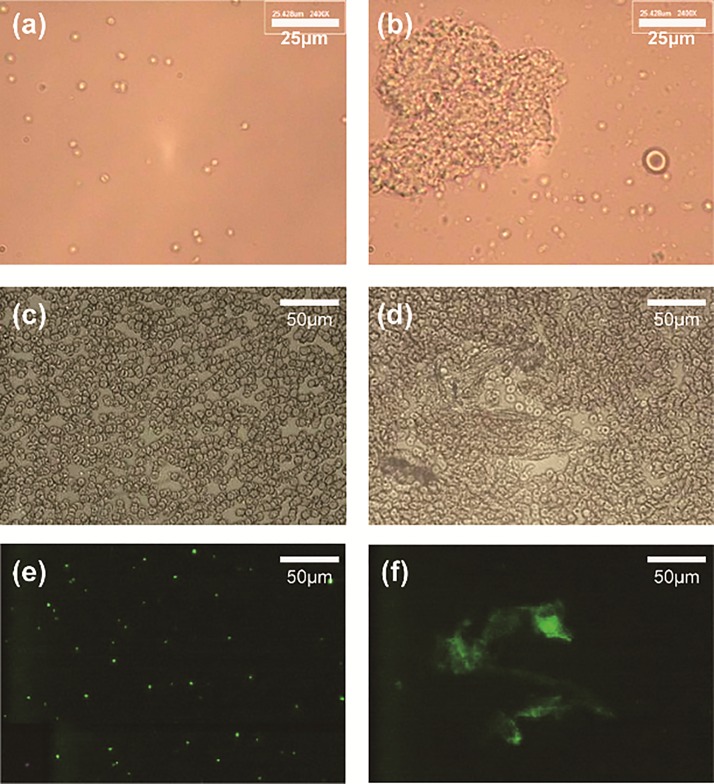
Images of platelet aggregation by shear stimulation with a rotating stirrer at 2800 rpm for 180 s in PRP and whole blood. (a) and (b) show platelets in PRP in a bright field after stirring at 0 and 2800 rpm, respectively. The scale bar indicates 25 *μ*m. (c) and (d) show bright-field images of whole blood after stirring at 0 and 2800 rpm, respectively. (e) and (f) show fluorescent images for the control and sheared sample at 2800 rpm, whose locations are coincident with (c) and (d), respectively. The scale bar indicates 50 *μ*m.

**FIG. 4.**
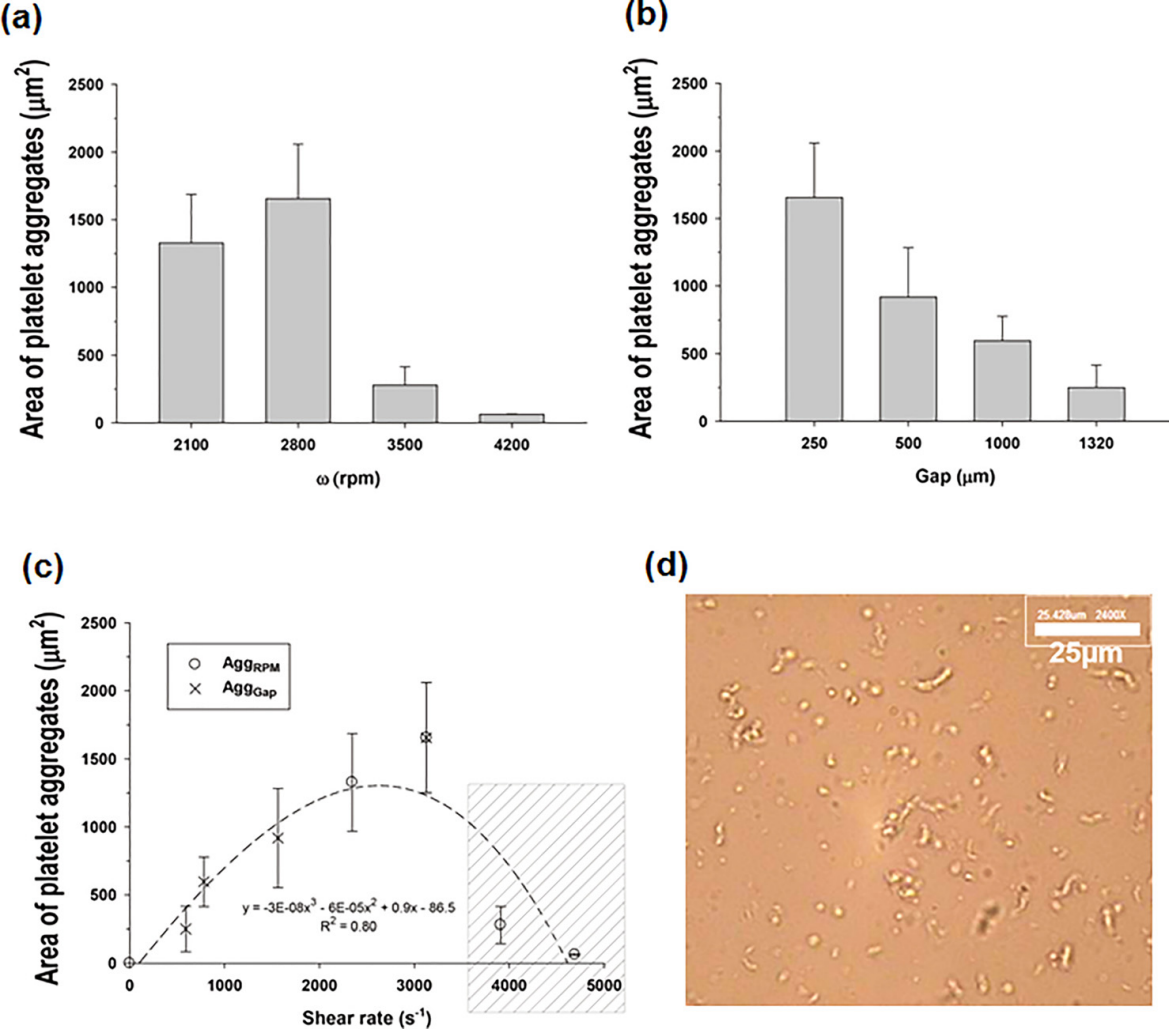
Bar plot of the area of platelet aggregates in PRP by shear stimulation regulated with different *ω* and *h*. (a) shows the area of the platelet aggregates with respect to the rotational speed of the stirrer for a 1× bar stirrer with a gap of 250 *μ*m. (b) shows the area of the platelet aggregates with respect to the gap between the stirrer top and chamber top for a for a 1× bar stirrer at 2800 rpm. (c) re-arranges the results according to the shear rates, which were calculated using Eq. (2) by using the *ω* and *h* changes in (a) and (b), respectively. (d) is an example of the platelets sheared at 4700 s^-1^ in the box shaded with diagonal lines in (c), which exhibits less-aggregated platelets. The scale bar indicates 25 *μ*m.

The rotating stirrer in the confined chamber produced a significant activation of the platelets, causing them to aggregate. Normal platelets in the PRP were individually dispersed, as shown in Figure 3(a). After a shear stress was induced using a stirrer rotating at 2800 rpm for 180 s, the platelets were activated and became a platelet aggregate, as shown in Figure 3(b). This platelet aggregate, for which the maximum diagonal length was 80 *μ*m, was far larger than the erythrocyte shown in the right side of the picture. Figures 3(d) and 3(f) show platelet aggregation in whole blood under the same shearing conditions as those shown in Figure 3(b). Platelet aggregates that could barely be distinguished among the blood cells shown in Figure 3(d) can be clearly observed in the fluorescent-field image with DIOC_6_ staining in Figure 3(f). Furthermore, as discussed in our previous study (Ref. 19), activated platelets were counted using flowcytometry. More than 87% of the activated platelets used 5× bar for 30 s at 2800 rpm, which are not shown. These analyses are summarized in Figure 4.

Figure 4 shows the average area of the platelet aggregates formed by shear stimulation with a rotating stirrer for PRP. In this experiment, a narrow stirrer was employed. Platelet aggregation depended strongly on the stirring speed (*ω*) and flow gap (*h*). As the stirring speed *ω* was increased, the area of platelet aggregation also increased, but this also tended to rapidly decrease beyond 2800 rpm, as shown in Figure 4(a). In Figure 4(b), the area of the platelet aggregates exponentially decreased as *h* increased. These results, which are related to the controllable parameters *ω* and *h*, were rearranged according to the average shear rate ($\dot{\gamma }$), as shown in Figure 4(c). The area of the platelet aggregates increased with the shear rate until the criterion for the shear rate—approximately 3100 s^−1^—was satisfied. However, a drastic decrease in aggregation occurred when the criterion was exceeded.

In previous studies, the threshold shear rate required to activate platelets was found to be 5000 s^−1^ or far higher (∼10 000 s^−1^).^5,29^ The difference in the threshold shear rates required to activate the platelets might be a result of the different definitions of the shear rate among the studies. The threshold shear rate in the previous study^12,13,15^ was defined at the wall, considering that the platelets in a circular duct migrate outwards in the radial direction.^30,31^ However, the radial migration velocity is relatively small compared to main velocity and thus it takes a relatively long time and a sufficient flow length. Therefore, the platelets in a pressure-driven flow system may not be exposed to a uniform shear rate.

In a geometry consisting of the present stirring system, the shear rate is not uniform as in the case of a parallel-disc rheometry. However, there is a significant secondary flow, which facilitates the migration of the platelets to every radial position, therefore exposing all the shear fields in the chamber. A platelet located at an arbitrary radial position gradually migrates inwards in a spiral pattern and then rapidly moves outwards because of the rotating stirrer motion. Thus, all the platelets are effectively and completely exposed to a range of shear rates because of the secondary flow in the chamber.

**FIG. 5.**
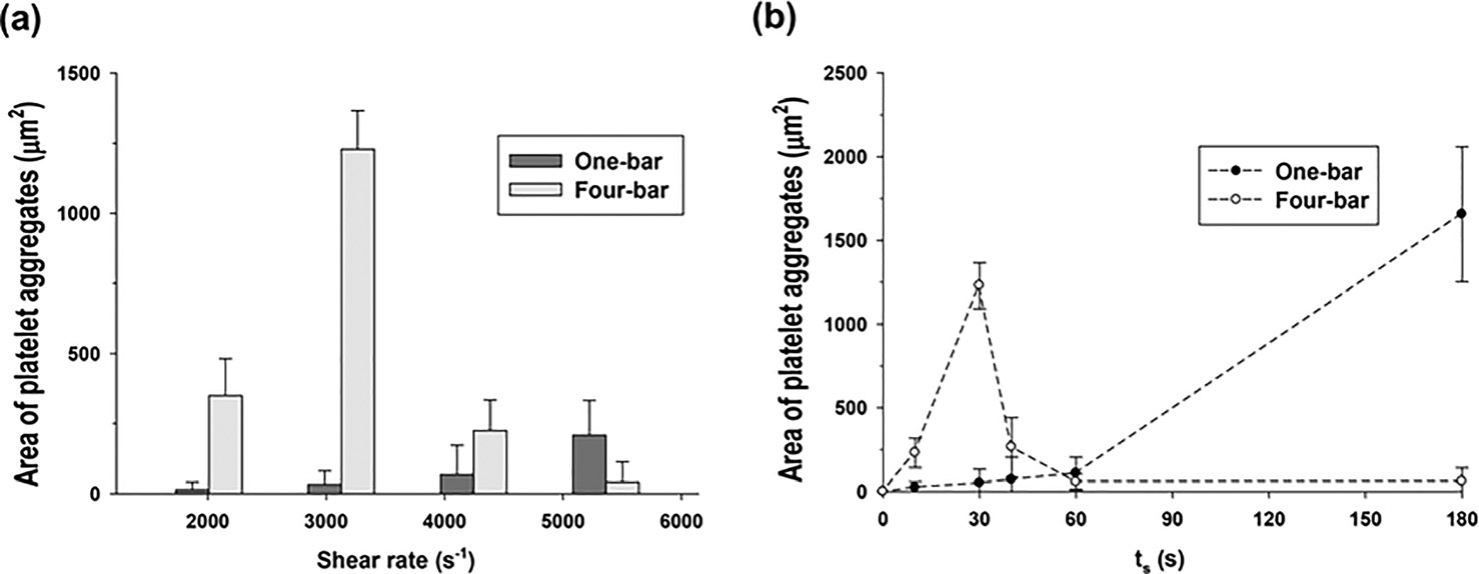
Effect of the surface area of the stirrer on platelet aggregation. Areas of platelet aggregates induced by one-bar and four-bar stirrers are compared. (a) indicates the area of the platelet aggregates with respect to *ω* during a fixed period of *t_shear_* = 30 s. (b) shows the area of the platelet aggregates induced by *t_shear_* at a fixed *ω* of 2800 rpm (shear rate of 3100 s^−1^).

In the present study, we identified an innovative rotating-stirrer design that can produce quick and efficient mixing as well as effective shear generation. The key design parameter was the surface area of the stirrer (*A_stirrer_*). For the two different stirrer widths (1 and 4 mm), platelet aggregation was measured with a fixed shearing time (30 s), as shown in Figure 5(a). The narrow and wide stirrers yielded maximum platelet aggregation at shear rates of 5300 and 3100 s^−1^, respectively. For the narrow stirrer, the platelets were not fully aggregated after 30 s. The wide stirrer produced the maximum area of platelet aggregation at a moderate shear rate (3100 s^−1^) after a short shearing time.

The effect of the shearing time (*t_shear_*) on platelet aggregation was examined for a fixed shear rate of 3100 s^−1^. Surprisingly, the wide and narrow stirrers induced maximum platelet aggregation at 30 s and 180 s, respectively, as shown in Figure 5(b). These results imply that the wide stirrer efficiently activated the platelets within a short shearing time. If the surface area of the stirrer is increased while the narrow flow gap remains fixed, the frictional force will be sufficient to move all of the fluid in a chamber such as a lumped fluid. Thus, a wide stirrer generates effective shear fields, which causes shear-induced platelet activation and subsequent platelet aggregation.

Furthermore, the common tendency of platelet aggregation decreases above the critical shear rate in Figures 4 and 5. Further application of a high shear rate to platelet aggregates forces them to disaggregate, as shown in Figure 4(d). If the binding force between platelets is greater than the cellular yield stress, then platelet aggregates are mechanically torn down with high shear rates.

**FIG. 6.**
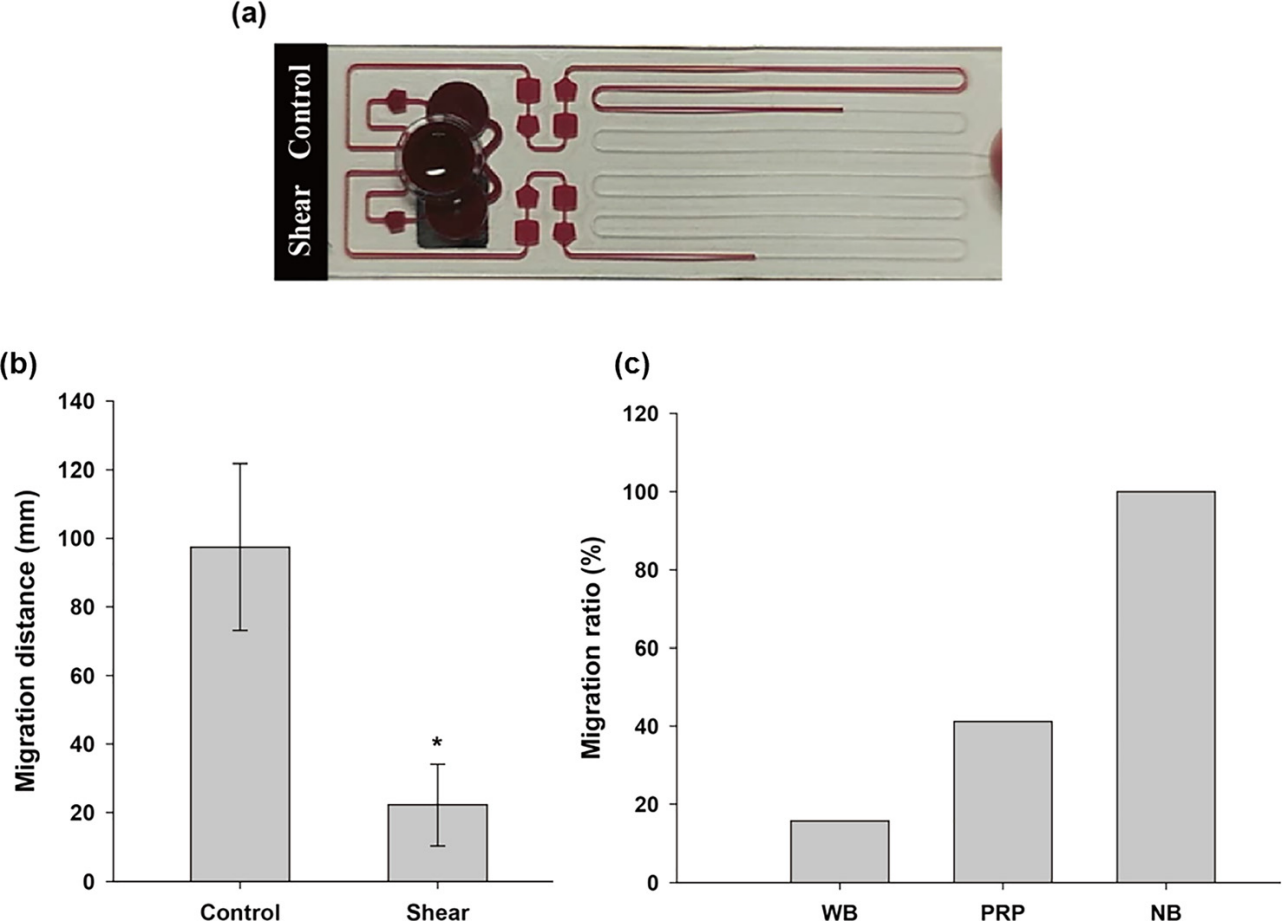
Difference in migration distance by stirring-induced platelet activation. (a) Photograph of the blood test using the microfluidic chip. (b) Migration distance in microfluidic channel from healthy donors (n = 3). * means p < 0.001. (c) Migration ratio according to the existence of the platelet and erythrocyte in the blood sample from a randomly selected healthy donor, where WB, PRP, and NB are whole blood, platelet rich plasma, and negative blood (i.e., not including platelets), respectively.

The optimal condition for platelet activation ($\dot{\gamma }$ = 3100 s^−1^ and *t_shear_* = 30 s with a four-bar stirrer) was examined for a microfluidic system. Figure 6(a) shows a photograph of a blood test using a microfluidic channel. A sheared blood sample flows less than the control. This clear difference in channel flows, which can reflect shear-induced platelet activation, was quantified by the MD. Figure 6(b) shows the distinct difference in the MD of the control and the sheared blood sample (n = 3). When the platelets are activated in a rotating shear flow, the MD of the sheared sample fell to one-fifth that of the control MD.

Figure 6(c) shows the effect of platelets and erythrocytes on channel blocking. Although erythrocytes are one of the most important components for thrombus formation in fibrin mesh, in primary hemostasis the platelets make a much greater contribution to rapid hemostasis *in vivo*.^32^ Similarly, the migration ratio (MR), which is the normalized MD of a sheared sample by the control, can reflect the major contribution of the platelets in the early stages of hemostasis *in vitro*. While whole blood (WB) showed a significantly small value of MR of around 20%, NB reached the final end point without stopping. Furthermore, as erythrocytes can easily deform and pass though small capillaries,^33,34^ they would easily flow through the micro channels. However, even though PRP has a much lower viscosity relative to WB, the PRP sample eventually stopped with a fairly lower MR value than that for NB. Although the erythrocytes indirectly help to inhibit sample flow, it is noteworthy that the MD is highly dependent on platelet activation.

We investigated the platelet activation with a rotating-stirrer in a microchamber. Platelets were effectively and quickly activated using the present stirring system. Platelets exposed to shear stress even for a short time were irreversibly activated and aggregated. The wide stirrer significantly shortened the shearing time required to activate the platelets and realized the efficient mixing and subsequently homogeneous activation of the platelets with a strong secondary flow. The degree of platelet activation was also correlated with the platelet-induced flow stoppage by using a microfluidic test system. Thus, the stirrer-chamber system facilitates quick, simple, and accurate shear-dependent platelet function tests because of its effective shear generation.
